# Effect of patient Age on surgical outcomes for Graves’ disease: a case–control study of 100 consecutive patients at a high volume thyroid surgical center

**DOI:** 10.1186/1687-9856-2013-1

**Published:** 2013-01-25

**Authors:** Christopher K Breuer, Daniel Solomon, Patricia Donovan, Scott A Rivkees, Robert Udelsman

**Affiliations:** 1 Department of Surgery, Yale University School of Medicine, New Haven, CT, US; 2 Department of Pediatrics, Yale University School of Medicine, New Haven, CT, US; 3 Yale Pediatric Thyroid Center, Yale University School of Medicine, New Haven, CT, US

**Keywords:** Hyperthyroidism, Thyroidectomy, Graves’s disease, Thyroid, Pediatric, Child, Adult

## Abstract

**Objective:**

To compare outcomes between children (<18 yrs) and adults undergoing total thyroidectomy for Graves’ disease (GD) at a high volume, multidisciplinary thyroid center.

**Summary of background data:**

Reported complication rates for children undergoing surgery for Graves’ disease are worse than for adults.

**Methods:**

100 consecutive patients (32 children; 68 adults) who underwent total thyroidectomy for Graves’ disease (GD) by a high-volume endocrine surgery team from were compared.

**Results:**

The mean patient age was 9.7 yrs (range 3.4-17.9 yrs) in children versus 44.9 yrs (range 18.4-84.2 yrs) in adults. Operative times were longer in children (2.18 ± 0.08 hrs) than in adults (1.66 ± 0.03 hrs) (p = 0.003). Pediatric thyroid specimens averaged 38.6.0 ± 8.9 gm (range: 9–293 gm) and adult thyroid specimens averaged 48.0 ± 6.4 gm (range: 6.6-203 gm) (p = 0.34). Thyroid to body weight ratios were greater in children (0.94 ± 0.11 gm/kg) than adults (0.67 ± 0.8 gm/kg) (p = 0.05). In all patients, the hyperthyroid state resolved after surgery. There was no operative mortality, recurrence, or permanent hypoparathyroidism. Transient post-operative hypocalcemia requiring calcium infusion was greater in children than adults (6/32 vs. 1/68; p = 0.004). Transient recurrent laryngeal nerve dysfunction occurred in two children and in no adults (p = 0.32). Postoperative hematoma occurred in two adults and in no children (p = 0.46). The length of stay was longer for children (1.41 ± 0.12 days) than for adults (1.03 ±0.03 days) (p = 0.004).

**Conclusion:**

Surgical management of GD is technically more challenging in children as evidenced by longer operative times. Whereas temporary hypocalcemia occurs more commonly in children than adults, the risks of major complications including disease recurrence, permanent hypoparathyroidism, recurrent laryngeal nerve injury, or neck hematoma were indistinguishable. These data suggest that excellent and equivalent outcomes can be achieved for GD surgery in children and adults when care is rendered by a high volume, endocrine surgery team.

## Introduction

A recent analysis of the Healthcare Cost and Utilization Project National Inpatient Sample (HCUP-NIS) database evaluated outcomes in children undergoing thyroid and parathyroid surgery [1]. Pediatric patients were observed to have higher complication rates than those reported for adults [1,2]. Pediatric complication rates were lower when the surgery was performed by a high volume endocrine surgeons, defined as performing >30 cases per year [3-5], but remained higher than those reported for adults [1,3,6].

We hypothesized that an endocrine surgical team consisting of a high volume endocrine surgeon, a pediatric surgeon, a pediatric endocrinologist and an experienced endocrine surgery nurse could attenuate the outcome gap between pediatric and adult surgical patients. To evaluate the outcome of this approach, we compared the outcomes of 100 consecutive pediatric and adult patients who underwent thyroidectomy for Graves’ disease (GD) at a single institution by high volume adult and pediatric endocrine surgery team.

## Methods

The Yale Pediatric Thyroid Center is a multidisciplinary team composed of a high volume endocrine surgeon (greater than 200 thyroidectomies/year), a pediatric surgeon, pediatric endocrinologists, pediatric anesthesiologists and experienced nursing and supportive staff. The Yale Pediatric Thyroid Center sees about 300 pediatric patients per year with thyroid disorders and approximately 30 pediatric patients undergo thyroid surgery annually. All pediatric patients who underwent total thyroidectomy for GD as part of the Yale Pediatric Thyroid Center between June 2002 and November 2010 were entered into a database. Adult patients operated on over the same period were identified, as well from CPT codes (60240, 60252). All pediatric procedures were performed by both attending surgeons. All but one patient were treated pre-operatively with supersaturated potassium iodide (SSKI) for at least 5 days prior to surgery. The one pediatric patient not treated fully with SSKI developed an allergic reaction after two doses and the medication was discontinued.

Twenty pediatric patients were treated with 0.5 mcg of calcitriol twice daily for 3 days before surgery. Post-operatively the calcitriol was weaned over 15 days (0.5 mcg bid × 5 days; then 0.5 mcg qd × 5 days; then 0.5 mcg qod for 5 days).

In all cases either a total or bilateral near-total thyroidectomy was performed. A total thyroidectomy was defined as a complete resection of the thyroid gland via an extracapsular dissection [7,8]. If it is was determined intraoperatively that a complete extracapsular dissection would likely result in irreversible damage to either the recurrent laryngeal nerve (RLN) or parathyroid gland(s), the capsule was entered and a miniscule amount of thyroid tissue was left in situ to avoid injury to either the RLNs or parathyroid glands, a procedure referred to as a near-total thyroidectomy [7,8].

All patients were admitted post-operatively. Serum calcium levels were measured every 4 to 8 hrs after surgery in the pediatric patients and in all patients on the morning of post-operative day one. Intravenous calcium gluconate therapy was administered if serum calcium levels (corrected for total protein) measured <7.5 mg/dL without symptoms of hypocalcemia or measured <8.0 mg/dL in the presence of symptomatic hypocalcaemia (e.g. hand parasthesias, perioral numbness, muscle cramps, the presence of a Chvostek’s sign).

Pre-operative demographics, comorbidities and medications were recorded. Operative details including detailed contemporaneous operative illustrations, post-operative course, serum calcium levels and operative pathology were recorded. Permanent recurrent laryngeal nerve (RLN) injury, hematoma and permanent hypoparathyroidism were classified as major complications. Transient hypocalcemia requiring intravenous calcium infusion and transient RLN neuropraxia were considered to be minor complications.

Statistical analysis was performed using Students’t-test and Fisher’s exact test as appropriate. Statistical significance was determined when p < 0.05. Values shown are mean ± standard deviation.

The Yale Human Investigations Committee approved this study.

## Results

Thirty two children and 68 adults underwent total or near-total thyroidectomy. The clinical characteristics of the patients are described in Table 1. The mean age at time of surgery was 9.7 years (range 3.4-17.9 yrs) for children and 44.9 years (range 18.4-84.2 yrs) for adults. Females constituted 81.3% of the children and 86.7% of the adult patients.

**Table 1:** Clinical characteristics of adult and pediatric patients

	**Adult**	**Pediatric**	**p-value**
Patients	68	32	
Patients requiring IV Calcium	1	6	0.004*
RLN Injuries	0	1	0.32*
Hematomas	2	0	0.46*
Incidental Cancers	12	1	0.01*
Thyroid mass (g)/Body mass (kg)	0.67	0.94	0.05^†^
Operative Time (hour:min)	1:37	2:09	0.001^†^
Inpatient Post Operative Days	1.03	1.41	0.004^†^

* - Fisher's Exact test.

^†^ - Student's *t*-test.

Pediatric procedures required longer operative time than those of adults (1.18 ± 0.08 hrs vs. 2.09 ± 0.03 hrs, p = 0.003). Pediatric thyroid specimens averaged 38.6.0 ± 8.9 gm (range 9–293 gm) (p = 0.34) and adult thyroid specimens averaged 48.0 ± 6.4 gm (range 6.6-203 gm) (p = 0.34). When the thyroidectomy specimen weight was considered in relation to that of the patient, the pediatric patients had larger glands per kilogram of body mass as compared to the adults (0.94 ± 0.11 gm/kg vs. 0.66 ± 0.07 gm/kg, p = 0.05)

Pathological analysis of the operative specimens revealed occult malignancy in one of the 32 pediatric patients and in 12 of the 68 adult patients (p = 0.03). The one pediatric patient had a papillary microcarcinoma of 0.4 cm in greatest diameter confined to the thyroid capsule. In the adults, 10 of 12 malignancies represented papillary microcarcinomas that were less than 0.3 cm in greatest diameter. One case was a multifocal papillary carcinoma that included a papillary carcinoma that was 2.5 cm in greatest diameter. There was one Hürthle cell adenoma in an adult patient.

Pediatric patients were more likely to require postoperative intravenous calcium infusion than adults (18.0% vs. 1.4%, p = 0.004). Pediatric patients required a longer length of stay than adults (1.41 ± 0.12 vs. 1.03 ±0.03 days, p = 0.004) reflecting the greater need for calcium infusions.

When preoperative calcitriol was not given 50% of pediatric patients required calcium infusion with a mean duration of infusion of 1.5 ±0.3 days. When calcitriol was given, 16% of patients required calcium infusion with a mean duration of infusion of 1.25 days (p 0.02 vs. no caclitriol).

There was one case of transient RLN neuropraxia in a child that recovered within 6 months after surgery. One child sustained a RLN transaction during surgery that was recognized and repaired during the initial procedure. Long term follow-up revealed that RLN function was restored as evidenced by bilateral vocal cord mobility 12 months after surgery. There were no RLN injuries in the adults.

There were 2 post-operative hematomas in the adult group requiring operative exploration and none in the pediatric group (p = 0.46). There was no operative or perioperative mortality, and no cases of permanent hypoparathyroidism. Follow up data indicated no recurrences of hyperthyroidism in either children or adults over up to a maximal follow-up period of 8 years. The overall rates of major complications were indistinguishable comparing children and adults.

## Discussion

Graves’ disease is rare in children with a prevalence of 1 in 10,000 [9,10]. Because only a minority of pediatric patients achieve remission [11-14], the majority of patients will require either radioactive iodine or surgery [4,9]. Our observations support the notion that in the hands of high volume thyroid surgeons, thyroidectomy is an appropriate treatment option for pediatric patients.

To date the largest series detailing the surgical treatment of GD in children is from the Mayo Clinic [15]. Seventy-eight pediatric patients, ages 3.1 to 17.9 years, with GD underwent surgical treatment [15]. Sixty patients (77%) underwent total or near-total thyroidectomy; 18 patients (23%) underwent bilateral subtotal thyroidectomy [15]. When a sub-total thyroidectomy is performed, a small amount of thyroid tissue is left behind in the region of the RLN or parathyroid glands in the hope of minimizing risks of RLN injury or hypoparathyroidism [8].

In the Mayo Clinic series, no RLN injuries, hematomas, nor instances of permanent hypoparathyroidism were reported [15]. One patient (1%) developed transient RLN neuropraxia, and 5 patients (6%) had transient hypoparathyroidism [15]. Five microcarcinomas (6%) were identified in the pathological specimens [15]. Of note, 4 of the 18 patients (22%) who underwent bilateral subtotal thyroidectomy experienced recurrence of their hyperthyroidism [15].

Recognizing the high recurrence rate associated with subtotal thyroidectomy, we adopted an aggressive surgical approach for the management of GD in children and adults by performing total or near-total thyroidectomies in all patients. Supporting the utility of our approach in curing the disease, no patient in our series experienced relapse of their GD.

HCUP-NIS data show that pediatric patients suffer complications more frequently following thyroidectomy than adults (9.3% vs. 6.1%, p < 0.01) [1,2]. In our cohort, we observed transient hypocalcemia, presumable due to transient hypoparathyroidism, as the most common minor complication in pediatric patients. However, with preoperative calcitriol therapy, the need for post-operative calcium infusions was reduced from 50% to 16% and the duration of intravenous calcium infusion was shortened by more than 50%.

RLN injury was observed in two of the children in our cohort. In one child, a soft voice was observed shortly after surgery, and direct laryngoscopy performed two months after surgery revealed unilateral vocal cord paresis. At six months after surgery, there was normal bilateral vocal cord function and the voice was normal.

One child, 4 years of age, had transection of one RLN during surgery that was immediately recognized and repaired. At 6 months after surgery, unilateral vocal cord paresis was observed by direct laryngoscopy. At 18 months after surgery, direct laryngoscopy revealed bilateral vocal cord movement and the voice was normal. Of note, the RLN injury occurred in the lone patient who could not be treated preoperatively with SSKI due to an anaphylactic reaction to the medication.

It is interesting that occult malignancy was seen in 12 of 68 adult patients and in 1 of 32 pediatric patients. Ten of the 12 malignancies seen in adults were unifocal papillary microcarcinomas, and one case was a multifocal papillary carcinoma that was 2.5 cm in the greatest diameter. A papillary microcarcinoma of 0.4 cm confined to the thyroid capsule was seen in one pediatric patient. These observations highlight the need for clinicians to obtain sonographic imaging of the thyroid gland if asymmetry or gland size change is noted in the setting of GD. Should nodules be observed, they should be evaluated per recent guidelines [16].

The observation of microcarcinomas in the setting of GD, which will be difficult to identify by preoperative ultrasound, should not be construed as an argument to in favor of surgery over radioactive iodine in the definitive treatment of GD. If GD patients with microcarcinomas are treated with recommended activities of radioactive iodine that are intended to ablate thyroid tissue [4,17,18], it is anticipated that microcarcinomas will be destroyed along with normal thyroid tissue. Support for this notion come from data from the Thyrotoxicosis Study Group follow-up data showing that rates of differentiated thyroid cancer in patients with GD are substantially lower in those treated with radioactive iodine or surgery than in individuals treated with antithyroid medications alone who underwent spontaneous remission [19].

Overall, we demonstrate that total or near-total pediatric thyroidectomy can be performed safely in experienced hands and is not associated with GD recurrence. A multidisciplinary team consisting of a high volume endocrine surgeon, a pediatric surgeon, pediatric endocrinologists, pediatric anesthesiologists, and experienced endocrine nurses can achieve excellent outcomes with very low complication rates. These observations support recent recommendations that children and adults requiring surgery for GD should be operated on by high volume endocrine surgical teams [4,5].

## Competing interests

The authors declare that they have no competing interests.

## Authors’ contributions

SR, CB conceived and participated in the design of the study. RU, CB and SR performed the surgeries. DS and PD participated in the sequence and coordination of the study. SR and RU helped draft the manuscript. All authors read and approved the final manuscript.
